# Membrane interaction to intercellular spread of pathology in Alzheimer’s disease

**DOI:** 10.3389/fnins.2022.936897

**Published:** 2022-09-09

**Authors:** Deepak Kunhi Valappil, Neeraj Jayakumar Mini, Aysha Dilna, Sangeeta Nath

**Affiliations:** Manipal Institute of Regenerative Medicine, Manipal Academy of Higher Education, Manipal, India

**Keywords:** Alzheimer’s disease, neurodegenerative diseases, prion-like propagation, exosomes, tunneling nanotubes (TNTs), intercellular communication, amyloid-β, tau

## Abstract

Progressive development of pathology is one of the major characteristic features of neurodegenerative diseases. Alzheimer’s disease (AD) is the most prevalent among them. Extracellular amyloid-β (Aβ) plaques and intracellular tau neurofibrillary tangles are the pathological phenotypes of AD. However, cellular and animal studies implicate tau as a secondary pathology in developing AD while Aβ aggregates is considered as a trigger point. Interaction of Aβ peptides with plasma membrane (PM) seems to be a promising site of involvement in the events that lead to AD. Aβ binding to the lipid membranes initiates formation of oligomers of Aβ species, and these oligomers are known as primary toxic agents for neuronal toxicities. Once initiated, neuropathological toxicities spread in a “prion-like” fashion probably through the mechanism of intercellular transfer of pathogenic aggregates. In the last two decades, several studies have demonstrated neuron-to-neuron transfer of neurodegenerative proteins including Aβ and tau *via* exosomes and tunneling nanotubes (TNTs), the two modes of long-range intercellular transfer. Emerging pieces of evidence indicate that molecular pathways related to the biogenesis of exosomes and TNTs interface with endo-lysosomal pathways and cellular signaling in connection to vesicle recycling-imposed PM and actin remodulation. In this review, we discuss interactions of Aβ aggregates at the membrane level and its implications in intercellular spread of pathogenic aggregates. Furthermore, we hypothesize how spread of pathogenic aggregates contributes to complex molecular events that could regulate pathological and synaptic changes related to AD.

## Graphical Abstract

APP (amyloid precursor protein) cleavage at plasma membrane (PM) generates Aβ peptides extracellularly. Interaction of Aβ peptides with PM promotes toxic amyloid aggregates. PM damage induced by toxic aggregates facilitate internalization and accumulation of Aβ to Multi Vesicular Bodies (MVB) and lysosomes, and proteotoxicity stress could subsequently modulate intercellular spreading of pathogenic aggregates in AD *via* exosome release and TNTs (tunneling nanotubes). Progression of Aβ toxicities may facilitate tau toxicities and formation of Neuro Fibrillary Tangles (NFT) in AD. 
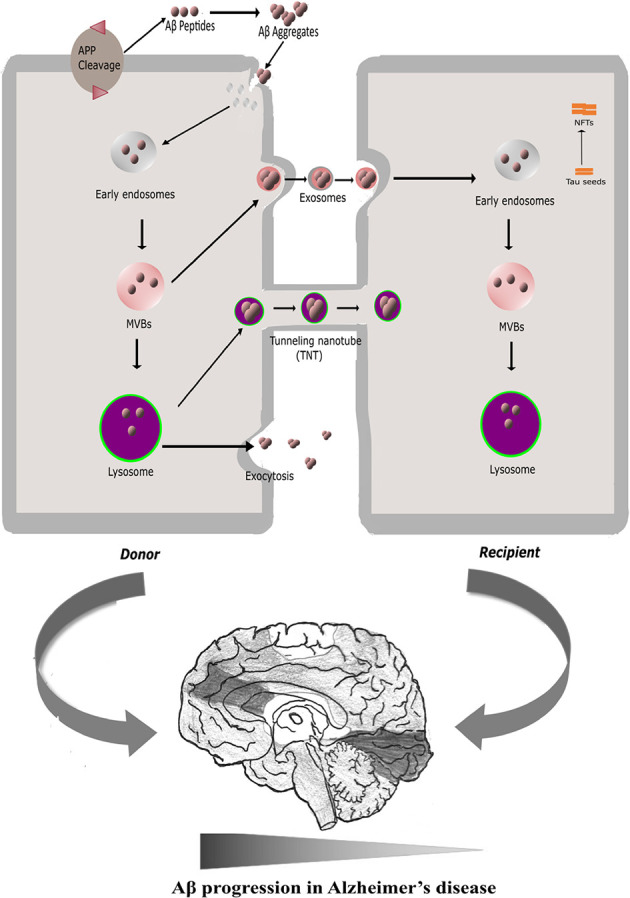

## Introduction

Extracellular Aβ plaques and intracellular tau neurofibrillary tangles (NFTs) are the pathological phenotypes of AD. APP (amyloid precursor protein) cleavage at plasma membrane (PM) of neuronal cells results in amyloid-β (Aβ) peptide generation extracellularly, following which these peptide aggregate and gradually form fibrils or plaques (Ito et al., 2011; Zhang et al., 2011). Later, several studies have stated that the soluble oligomers are explicitly neurotoxic and intraneuronal Aβ accumulation acts as a disease driver (McLean et al., 1999; LaFerla et al., 2007; Gouras et al., 2010). However, contributions of extracellular deposits and intraneuronal accumulation of Aβ in AD pathogenesis is complex and contradictions were reported (McLean et al., 1999; LaFerla et al., 2007; Gouras et al., 2010). The study (Bloom, 2014) has shown that intraneuronal Aβ accumulation plays no significant role to form NFTs. On the other hand, several studies have shown that human subjects with amyloid deposits and senile plaques in the brain display no cognitive impairment (Andersen et al., 2017; Kametani and Hasegawa, 2018; Morris et al., 2018). Clinicopathologic studies on human subjects found that cognitive symptoms vary between patients with temporal patterns of deposits (Garcia-Marin et al., 2009; Blazquez-Llorca et al., 2010; Nelson et al., 2012). Studies have also shown that, neuropathological changes in brain areas vary within the same patient (Garcia-Marin et al., 2009; Blazquez-Llorca et al., 2010; Nelson et al., 2012). The spreading of neuropathology and cognitive impairment follows different temporal patterns depending on the different regions of the brain from which the pathologies have begun to spread (Braak and Braak, 1991; Thal et al., 2002; Jack et al., 2019).

Mutations that enhance the aggregation propensity of Aβ or changes its production, processing, and clearance are implicated in AD (Oddo et al., 2006). The membrane environment can enhance the transition of monomeric Aβ to the toxic β-sheet conformation, which acts as a nucleation location for rapid self-aggregation, to form pre-fibrillar oligomers and eventually the fibrils, depending on the lipid component inside the membrane (Yasumoto et al., 2019). Oligomers induce a toxic cascade involving PM damage (Yasumoto et al., 2019), lipid oxidation, ion-permeable membrane pores, as a result of which internalization takes place in the vesicles of endo-lysosomal pathways (Sengupta et al., 2016; Julien et al., 2018; Dilna et al., 2021).

Another pathological feature of AD is the endo-lysosomal dysfunction, which is induced by toxic Aβ aggregates (Murphy and LeVine, 2010; Whyte et al., 2017). This dysfunction alters the trafficking of multivesicular bodies (MVBs) to lysosomes or to PM (Baixauli et al., 2014), thereby, enhances exosome release. In 1980s, exosomes were identified in the extracellular space as one of the subtypes of extracellular vesicles (EVs) (Johnstone et al., 1987). Studies have shown that exosomes are key players in the transport of molecules and cell-to-cell spreading of pathology in neurodegenerative diseases (Howitt and Hill, 2016). Exosomes isolated from the brain of AD patients contained elevated levels of Aβ oligomers, which vehicle the spread of Aβ pathology from one neuron-to-another (Sardar Sinha et al., 2018). Similarly, tau also spreads *via* exosomes and elevated levels of tau within exosomes were also detected in the cerebrospinal fluid (CSF) of AD patients (Wang et al., 2017).

Recent discoveries revealed that cells establish essential long-range intercellular communications by tunneling nanotubes (TNTs) (Raghavan et al., 2021). TNTs are long-range intercellular membrane-actin conduits (Rustom, 2004), reported to be responsible for the direct transfer of organelles, neurodegenerative aggregates (Victoria and Zurzolo, 2017), pathogens like viruses (Jansens et al., 2020), and RNA between neighboring cells (Haimovich et al., 2021). TNT is defined as an open-ended actin membrane conduit that transfer organelles and pathogens directly between distant cells (Rustom, 2004). Cell-to-cell spreading of prion *via* TNTs was first demonstrated in 2009 (Gousset and Zurzolo, 2009). Then, studies have shown that almost all known neurodegenerative proteins (including Aβ and tau) use TNTs as their mode of intercellular transport to spread aggregates (Victoria and Zurzolo, 2017; Dilna et al., 2021; Zhang et al., 2021). Recently Dilna and collaborators (Dilna et al., 2021) have shown that cells repair PM perforation induced by oligomers of Aβ_1–42_, which promotes biogenesis of TNTs and cell-to-cell transfer. Thus, we discuss here toxic amyloid aggregates induced membrane disorder to subsequent endo-lysosomal dysfunctions and how they modulate various cellular signaling and intercellular spreading of pathogenic aggregates in AD (Graphical Abstract).

## Amyloid-β oligomers and membrane interactions

Amyloid-β peptides are generated from the transmembrane protein APP, by the sequential cleavage of β and γ secretases. Processing of transmembrane domain of APP by secretases produces N-terminal soluble domains sAPPα and sAPPβ, Aβ peptides and C-terminal fragments (Westmark, 2013). C-terminal cleavage between 40 and 42 hydrophobic residues is responsible for strong hydrophobicity in Aβ peptides (Nixon, 2017). The hydrophobic region is crucial in initiating the process of aggregation. The membrane environment can enhance the transition of hydrophobic Aβ peptides readily to the toxic β-sheet structure conformation, which acts as nucleation locations for rapid self-aggregation (Ito et al., 2011; Cline et al., 2018). Oligomerization may happen in several stages, the transformation of non-toxic monomers to oligomers and protofibrils can trigger neuronal damage (Hashimoto et al., 2003; Zempel et al., 2010). Aβ interacts with membrane and alters membrane compositions by binding to the surface and cell surface receptors such as APP, NMDAR and mGLuR5. Clustering of Aβ reduces lateral diffusion in the membranes and increases accumulation on excitatory synapses, resulting in synaptic dysfunction and LTD (long-term depression) (Westmark, 2013). Aβ increases membrane depolarization and Ca^2+^ influx (Blanchard et al., 2002). Aβ can also pervade the membrane and exert a wide range of toxicities to neurons, like functional alterations of neurons, dystrophy, neuronal loss, as well as synaptic dysfunctions (Niu et al., 2018). Pore/ion channel formation (Cline et al., 2018) and lipid extraction (Arispe et al., 1993; Delgado et al., 2016) are the major mechanisms which are present in the literature to explain the penetrating ability of Aβ.

## Amyloid-β-induced plasma membrane damage and repair

A leaky PM may be the result of a variety of events, like mechanical stress or pore-forming toxin attacks (Cooper and McNeil, 2015; Jimenez and Perez, 2017). It has already been shown that Aβ can form ion-permeable channels in a synthetic membrane. Aβ can induce a response to membrane repair similar to that induced by bacterial pore-forming toxin produced by *B. thuringensis* (Julien et al., 2018). The effects of Aβ oligomers, specifically tau hyperphosphorylation (Bilousova et al., 2016), can be mimicked by exposure to pore-forming toxins (Julien et al., 2018). Aβ oligomers induced PM damage and subsequently PM repair through coupled lysosomal exocytosis and endocytosis has recently been demonstrated (Dilna et al., 2021).

ESCRT (Endosomal Sorting Complex Required for Transportation) involved in the biogenesis of MVB, has also been shown to have a role in the PM repair (Jimenez et al., 2014). Aβ disrupts the PM integrity of the membranes which lack ESCRT-III machineries (Fruhmann et al., 2018). ESCRTs help in the APP sorting and accumulation of Aβ in MVBs (Edgar et al., 2015). Uptake of the exogenous Aβ oligomers leads to accumulation and enlargement of MVBs (Willén et al., 2017). The MVBs either are ubiquitinated for degradation, or targeted by lysosomes for their intracellular degradation, where they can be processed into perinuclear MVB, that are eliminated by exocytosis (Draeger et al., 2014). Recent studies have shown that ESCRT-III is not only involved in PM repair but also plays a significant role in early endo-lysosomal membrane repair (Radulovic et al., 2018).

## Cellular uptake and endo-lysosomal accumulation in Alzheimer’s disease

The intra-vesicular Aβ pool is generated from the secretase enzymes and APP, which are exclusively present in the lumen of the endo-lysosomal vesicles (Cataldo et al., 1997; Willén et al., 2017; Colacurcio et al., 2018). Accumulation of toxic pool of Aβ_1–42_ is evident in the endo-lysosomal vesicles when compared with Aβ_1–40_. Internalization of exogenous Aβ takes place by a mechanism of membrane stress sensitive, Rho GTPase regulated actin mediated endocytosis in neuronal cells (Wesén et al., 2017; Dilna et al., 2021). In primary neurons Aβ internalization takes place by a sphingolipid and cholesterol sensitive lipid rafts mediated dynamin dependent but clathrin independent pathway (Saavedra et al., 2007; Marshall et al., 2020). Internalization results in the selective accumulation of toxic pools of Aβ_1–42_ (Wesén et al., 2017). This could be a result of the non-degradable toxic pool that ultimately ends up accumulating in endo-lysosomal vesicles (Nath et al., 2012). Moreover, during PM repair toxin pores endocytose and traffic to lumen of MVBs and ultimately to lysosomes for degradation (Corrotte and Castro-Gomes, 2019).

## Potential modes of intercellular spread of pathology in neurodegenerative diseases

Prion-like cell-to-cell propagation of neurodegenerative proteins has been proposed since the discovery of prions. Li et al. (2008) has shown the trans-neuronal propagation of α-synuclein from grafted Lewy body neurons. Subsequently, several researchers demonstrated neuron to neuron transfer of neurodegenerative proteins α-synuclein (Desplats et al., 2009), tau (Clavaguera et al., 2009), polyglutamine (Ren et al., 2009), superoxide dismutase-1 (Ilieva et al., 2009), TDP-43 (Furukawa et al., 2011), and Aβ (Nath et al., 2012).

Aggregation-prone neurodegenerative proteins involved in AD, Parkinson’s disease (PD), Huntington disease (HD), Creutzfeldt-Jakob disease (CJD), and amyotrophic lateral sclerosis (ALS) were found to be contained within exosomes isolated from CSF and blood of patients with these conditions (Brettschneider et al., 2015). Prion-like proteins can be released by exosomes and internalized by the neighboring cells. This was proposed for Aβ (Rajendran et al., 2006), tau (Wang et al., 2017); α-synuclein (Fevrier et al., 2004; Danzer et al., 2007; Saman et al., 2012); superoxide dismutase (SOD-1) (Münch et al., 2011) and polyglutamine aggregates (Ren et al., 2009).

Spread of pathology *via* exosomes in neurodegenerative diseases has been widely studied, but the mechanisms by which neurodegenerative proteins propagate from one neuron to its neighboring one, is not completely studied yet. The recent studies of direct cell-to-cell transfer of prion-like proteins using TNT conduits have opened up a new avenue (Gousset et al., 2009). TNTs have been implicated in the intercellular transfer of almost all neurodegenerative proteins including, α-synuclein (Abounit et al., 2016; Valdinocci et al., 2021), mutant Huntington line (mHtt) (Costanzo et al., 2013), tau (Tardivel et al., 2016) and Aβ (Wang et al., 2011; Dilna et al., 2021; Zhang et al., 2021). It was shown that, the intracellular accumulation of neurodegenerative aggregates can induce biogenesis of TNTs and thereby the accumulated aggregates propagate from a cell to its neighboring one (Costanzo and Zurzolo, 2013; Abounit et al., 2016; Zhu et al., 2018).

## Modes of Alzheimer’s disease propagation through anatomical routes

The pathological progression of neurodegenerative disorders has been attributed to the prion-like self-propagation of toxic aggregates following the route of anatomically connected neurons (Prusiner, 2013). The transmission of AD pathology between neurons has been suggested (Lewis et al., 1987) since long, to explain the spread of pathology through anatomically connected brain regions. Inoculation of brain extract from AD patients manifests propagation of AD-like pathology in the brain of transgenic APP mice, but the propagation is not noticed in the non-transgenic control mice (Kane et al., 2000; Meyer-Luehmann, 2006). In addition, researchers have shown the propagation of synaptic dysfunction trans-synaptically from Aβ overexpressed neuron to its neighboring one (Harris et al., 2010; Wei et al., 2010).

The cell-to-cell transfer of tau (Clavaguera et al., 2009) and Aβ aggregates (Nath et al., 2012; Domert et al., 2014) have been demonstrated in *in vitro* neuronal models. Aβ induced pathology may directly or indirectly drive tau-mediated neurotoxicity and NFT formation (Blurton-Jones and Laferla, 2006). However, tau aggregates can propagate and induce other pathologies, independent of Aβ pathology (Victoria and Zurzolo, 2017). Therefore, understanding the neuron-to-neuron transfer of Aβ aggregates is highly demanding in the context of AD.

Exosomes isolated from AD patients were reported to be a potential vehicle which propagate Aβ aggregates from neuron-to-neuron (Rajendran et al., 2006; Sardar Sinha et al., 2018). Aβ oligomers when microinjected in a single hippocampal primary neuron spread through the neuronal path over time (Nath et al., 2012). A recent study has shown that crosstalk between astrocytes and glial co-culture *via* networks of TNTs resulted in increased degradation of Aβ aggregates *via* constant exchange of their internalized Aβ pools (Rostami et al., 2021). Studies have shown that extracellularly applied Aβ oligomers are internalized in the cell and then transferred between neuronal cells by lysosomal vesicles through TNTs (Wang et al., 2011; Dilna et al., 2021). It was also shown that, non-degradable aggregates transfer efficiently between neurons (Nath et al., 2012; Domert et al., 2014; Lim and Yue, 2015) and probably dominates the spreading through neuronal pathway over astrocytes or glial cells. A recent study has shown that tau and Aβ aggregates internalized from extracellular milieu transfer faster between cells through TNTs, compared to the intracellularly produced aggregates (Zhang et al., 2021).

## Endo-lysosomal pathology in the biogenesis of exosomes and tunneling nanotubes: The two long range intercellular transfer modes

Alterations in the endo-lysosomal pathway is another pathological hallmark of neurodegenerative diseases. Endo-lysosomal dysfunction regulates the trafficking of MVBs to lysosomes, PM (Baixauli et al., 2014) and regulate the release of exosomes. MVBs generate small intraluminal vesicles due to the inward budding of late endosomes. These bodies contain proteins, mRNA and lipids, which would either fuse with lysosomes for degradation or get released extracellularly after fusing with PM (Piper and Katzmann, 2007). The intracellular accumulation of amyloidogenic proteins aggravates endosomal abnormalities, lysosomal membrane damage and impair degradative capacity (Gauthier et al., 2017). Diminished degradative dysfunction can also induce a compensatory increase of exosome release (Alvarez-Erviti et al., 2011; Mathews and Levy, 2019).

Swollen lysosomes with accumulated Aβ aggregates were found at the axonal terminal surrounding the amyloid plaques in the AD brain (Domert et al., 2014; Gowrishankar et al., 2015). Large pools of Aβ accumulation were detected in both MVBs and lysosomes (Willén et al., 2017). MVBs accumulated with Aβ could enhance the spread of Aβ through exosome release (Rajendran et al., 2006). Exosomes isolated from the brain of AD patients contained high levels of Aβ (Sardar Sinha et al., 2018). The lysosomal stress associated with accumulation of amyloidogenic proteins contributes to biogenesis of TNTs (Abounit et al., 2016). Most of the studies have shown that appearance of TNTs is predominantly seen in case of cellular stress and diseased conditions. Studies indicated that the pathology associated with endo-lysosomal toxicities, vesicle recycling, and fusion with PM is involved in the formation of TNTs (Kimura et al., 2013; Victoria and Zurzolo, 2017).

Extracellularly applied Aβ oligomers induce PM damage and trigger the PM repair process *via* coupled endocytosis followed by lysosomal exocytosis (Julien et al., 2018). Our recent study found that Aβ oligomers induced PM damage. Subsequently, Ca^2+^ dependent membrane repair *via* lysosomal exocytosis instigates the biogenesis of TNT-like membrane nanotubes and cell-to-cell transfer of Aβ oligomers (Dilna et al., 2021). Similarly, extracellularly applied tau aggregates can induce the formation of TNTs in neuronal cells and tau proteins were found inside the TNTs (Abounit et al., 2016; Tardivel et al., 2016). The means of two extracellular release routes, lysosomal exocytosis and exosome release share a common interface within the endo-lysosomal system, but biogenesis of the two follows distinct pathways. Neurodegenerative aggregates induced alterations in endo-lysosomal pathway play a significant role to modulate the release of extracellular vesicles (Willén et al., 2017; Bécot et al., 2020).

Ras-related protein-A (RalA, small GTPase) promotes the biogenesis of TNTs by interacting with M-sec, a protein of exocyst complex (Hase et al., 2009). The release of exosomes and docking of MVBs are regulated by Rab 11 in a Ca^+2^ dependent manner (Escudero et al., 2014). Rab 11 plays an important role in vesicle recycling, inhibition of the same would interfere with membrane recycling and reduce the formation of TNTs (Zhu et al., 2018). Neurodegenerative proteins such as Aβ, α-synuclein, and mHtt, perturb Ca^2+^ homeostasis, and can deregulate lysosomal exocytosis (Freeman et al., 2013). Increased lysosomal exocytosis enhances the release of exosomes, probably by altering the preferential docking of MVBs to lysosomes, than fusing with PM (van de Vlekkert et al., 2019).

## Actin cytoskeleton regulatory pathways in the biogenesis of exosomes and tunneling nanotubes

Coordinated assembly and disassembly of actin filaments play a crucial role in regulated exocytosis. Actin cytoskeleton regulates integration of vesicles with PM and pre-fusion events in neurons, neuroendocrine, endocrine, and hematopoietic cells and regulates dynamics of extracellular vesicles release (Porat-Shliom et al., 2013). In other secretory cells, where large cargo molecules undergo slow exocytosis, actin plays a major role in post-fusion events by providing structural support to PM *via* curvature induced expulsion of large cargo (Porat-Shliom et al., 2013). Exosome biogenesis and sequential regulation can be controlled either in the endosomal pathway or at secretion. This coordination is achieved with the help of two small GTPases RhoA and Cdc42 (Chi et al., 2013). Cdc42, the effector molecule of p21-activated kinase (PAK), stimulates regulated exocytosis by activating actin polymerization, by modulating the downstream signals N-Wasp (Wiskott-Aldrich Syndrom Protein) and Arp2/3 (Gasman et al., 2003).

Interestingly, recent studies have shown that the Rho family of GTPases, Rac1, and Cdc42, play an important role in TNT formation in immune cells (Hanna et al., 2017). Similarly the endocytosis of HIV (human immunodeficiency virus) and HSV (herpes simplex virus) viruses *via* PAK dependent route induces the biogenesis of TNTs (Van den Broeke et al., 2009; Mukerji et al., 2012; Jacob et al., 2015; Jansens et al., 2020). Furthermore, cofilin an actin-binding protein, a downstream signaling cascade of PAK1, modulates F-actin polymerization and biogenesis of TNTs (Dagar et al., 2021). In our recent study, we have observed that the Aβ induced membrane damage instigates PAK1 dependent endocytosis and coupled lysosomal exocytosis to repair the membrane. The consequence of the repair mechanism promotes the formation of TNTs (Dilna et al., 2021).

## Actin regulatory pathways in intercellular spread and Alzheimer’s disease

Amyloid-β modulates activity of PAK pathways in several ways. Alteration of PAK pathways and consequent actin remodulations can lead to deep changes in neuronal health (Civiero and Greggio, 2018). However, role of PAK in AD pathogenesis is not clear. Aβ has been shown to modulate cofilin regulated actin polymerization through both PAK dependent and independent pathways. Aβ aggregate induced synaptic rigidity *via* cofilin pathology was reported by many studies (Kang and Woo, 2019). Presence of cofilin-actin rods/aggregates is a salient feature of AD (Bamburg and Bloom, 2009; Kang and Woo, 2019). Aβ aggregates intervene with various surface receptor mediated signaling cascades and promotes cofilin-actin rod formation by regulating the dephosphorylation of cofilin. Slingshot1 (SSH-1) is one of the conserved isoform of cofilin phosphatase, and Aβ induced activation escapes 14-3-3ζ mediated inhibition of SSH-1, which dephosphorylates cofilin.

**FIGURE 1:**
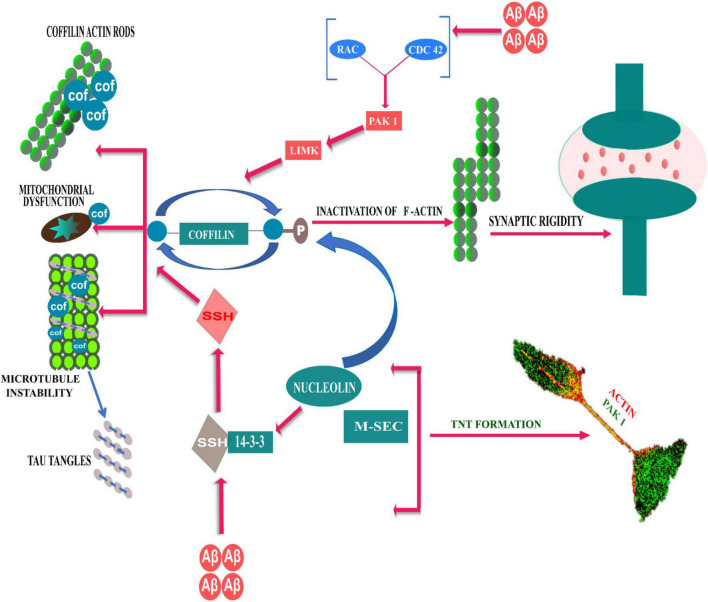
Schematic representative diagram to demonstrate Rho GTPases and actin cytoskeleton reorganization dynamics in TNT formation and AD development. Aβ modulates cofilin regulated actin polymerization through both PAK dependent and independent pathways. Slingshot1 (SSH-1) is one of the conserved isoform of cofilin phosphatase, and Aβ induced activation escapes 14-3-3ζ mediated inhibition of SSH-1, which dephosphorylates cofilin and resulting in cofilin-actin rod formation. On the other hand, Aβ aggregates can modulate LIM kinase mediated phosphorylation of cofilin *via* PAK kinase pathways (downstream of Rac/Cdc42), which can lead to over-activation of cofilin and synaptic dysfunction due to excessive actin dynamics. RNA-binding protein nucleolin interacts to M-sec, while nucleolin regulates 14-3-3ζ mRNA. The signaling axis of nucleolin and 14-3-3ζ mRNA phosphorylates cofilin to induce formation of TNTs.

On the other hand, Aβ aggregates modulate LIM kinase mediated inactivation/phosphorylation of cofilin, either by activating or inhibiting Rac/Pak signaling. PAK1, a Cdc42 GTP-bound effector, is a LIM kinase activator, that holds cofilin in an inactive state; hence, PAK1 depletion can lead to over-activation of cofilin and synaptic dysfunction due to excessive actin dynamics (Bamburg and Bloom, 2009). On contrary, cognitive deficits observed in AD models with Aβ-induced altered postsynaptic PAK levels and massive loss of postsynaptic protein Drebrin, could be prevented by an anti-Aβ antibody and/or by *in vivo* or *in vitro* PAK overexpression (Ma et al., 2008; Mokhtar et al., 2013). In addition, studies have shown that reduction of PAK1 in the cytosolic fraction, occurs due to aberrant activation and translocation of PAK to the membrane cytoskeletal fractions in the AD brain (Ma et al., 2008; Civiero and Greggio, 2018). From the literature it is not clear, how the role of activated PAK1 and its distributions in the cellular compartments affects the development of AD pathology.PAK1 plays an important role in cytoskeleton outgrowth and actin polarization in developing neurites (Daniels, 1998). Developing neurites connect to the neighboring astrocytes *via* TNT-like structures by gap junction mediated electrical coupling (Wang et al., 2012). In a cellular model of AD, we have recently shown that oligomeric Aβ_1–42_ induced phosphorylated active PAK1, promotes biogenesis of TNT-like structure for intercellular transfer of Aβ aggregates (Dilna et al., 2021). Role of cofilin has recently been reported in the biogenesis of TNT. A recent study by Dagar et al. (2021); suggests that, in addition to cofilin-regulated actin modulation, the exocyst complex protein, M-sec dependent recycling of membrane needs to function in a cohort, as a necessary step in the biogenesis of TNTs. RNA-binding protein nucleolin interacts with M-sec, while nucleolin regulates 14-3-3ζ mRNA, which is also required for the formation of TNT. On the other hand, Cdc42 and Rac1 (effector molecules of PAK) partially colocalize with exocyst complex and M-sec (Vitale et al., 2005; Lopez et al., 2008; Rondaij et al., 2008). However, further studies need to understand how oAβ induced TNT biogenesis *via* PAK1 activation pathway could contribute to development and progression of AD pathology. The role of regulatory signaling pathways of cofilin in TNT formation and AD is summarized here in the Figure 1 schematically.

## Discussion

Since decades, Aβ induced alterations and related toxicities at the membrane level are considered as a central cause for initiation of AD pathogenesis. Interestingly, Aβ induced membrane damage and repair, can direct endo-lysosomal accumulation to cell-to-cell spread of pathogenic aggregates *via* exosomes and/or tunneling nanotubes, the two long range intercellular modes of transfer. Spread of pathogenic aggregates could contribute to complex molecular events related to neuropathological and synaptic changes in AD.

Cells repair PM perforation induced by Aβ oligomers *via* Ca^+2^ dependent lysosomal exocytosis and coupled endocytosis *via* PAK1 dependent actin remodulation and this promotes biogenesis of TNTs and cell-to-cell transfer (Dilna et al., 2021). PM perforation by Aβ oligomers generates influx of intracellular Ca^+2^ and leads to synaptic dysfunction (Peters et al., 2016). Additionally, Aβ mediated alterations of PAK and cofilin phosphorylation play a vital role in synaptic dysfunction which leads to AD development. Interaction of Ca^2+^ with the membrane is a well-known phenomenon of membrane fusion, lysosomal exocytosis, and exosome release (Melcrová et al., 2016). Overall, the stresses which are induced by the PM damage and intracellular endo-lysosomal impairment aggravate the biogenesis of both exosomes and TNTs, and subsequently cell-to-cell transfer (Victoria and Zurzolo, 2017). Exosomes have also been demonstrated as a potential mediator to induce the formation of TNTs (Ady et al., 2014).

Alzheimer’s disease is categorically a disease characterized by degeneration of neurons, whereas astroglial cells and microenvironment of neurons play an important role in the disease progression. Several studies have shown that increased reactive astrocytes in the AD brain facilitate clearance of plaques and Aβ deposits (Frost and Li, 2017). The crosstalk between neurons and astroglial cells facilitates long-range intercellular transfer of neurodegenerative aggregates probably that helps to enhance cellular clearance of toxic burdens (Rostami et al., 2021; Scheiblich et al., 2021). On contrary, studies have also shown that reactive astrocytes enhance APP processing and as a result increased production of Aβ (Frost and Li, 2017). Reactive microglia have also been proposed to enhance propagation of tau pathology (Maphis et al., 2015; Hopp et al., 2018). Tau toxicity is also considered as one of the causatives of neurodegeneration. However, primary role of Aβ induced tau toxicity in AD pathology is debated. Several studies have shown that tau pathology can develop independent of Aβ induced toxicities and Aβ plays no role in developing tauopathies (Bloom, 2014).

In this context, it has to be mentioned here that AD confronts major challenges of variability in pathogenesis between patients. Variability of neurotoxicity in relation to membrane interactions with Aβ aggregates, have been studied a lot (Niu et al., 2018). Many diverse and complex interactions that exist between complex composition of membranes and transient Aβ aggregates are difficult to mimic and poorly understood. Thereby, to unfold the exact mechanism of progressive spreading in relation to Aβ aggregates induced membrane interactions and intercellular transfers, causes of variabilities need to be addressed. Nevertheless, revealing of the plausible mechanism of progressive spreading of AD pathology *via* intercellular transfer in association to PM damage, endo-lysosomal accumulation and actin remodulation may open up new inference in AD research.

## Author contributions

SN conceived the idea for the review. DV, NM, AD, and SN researched the literatures and wrote the manuscript. DV and NM drew the figures. All authors contributed to the article and approved the submitted version.
